# Direct PCR Offers a Fast and Reliable Alternative to Conventional DNA Isolation Methods for Gut Microbiomes

**DOI:** 10.1128/mSystems.00132-17

**Published:** 2017-11-21

**Authors:** Elin Videvall, Maria Strandh, Anel Engelbrecht, Schalk Cloete, Charlie K. Cornwallis

**Affiliations:** a Department of Biology, Lund University, Lund, Sweden; b Western Cape Department of Agriculture, Directorate Animal Sciences, Elsenburg, South Africa; c Department of Animal Sciences, Stellenbosch University, Matieland, South Africa; Vall d'Hebron Research Institute

**Keywords:** 16S rRNA, DNA extraction, direct PCR, library preparation, microbiota, repeatability

## Abstract

The gut microbiome of animals is emerging as an important factor influencing ecological and evolutionary processes. A major bottleneck in obtaining microbiome data from large numbers of samples is the time-consuming laboratory procedures required, specifically the isolation of DNA and generation of amplicon libraries. Recently, direct PCR kits have been developed that circumvent conventional DNA extraction steps, thereby streamlining the laboratory process by reducing preparation time and costs. However, the reliability and efficacy of direct PCR for measuring host microbiomes have not yet been investigated other than in humans with 454 sequencing. Here, we conduct a comprehensive evaluation of the microbial communities obtained with direct PCR and the widely used Mo Bio PowerSoil DNA extraction kit in five distinct gut sample types (ileum, cecum, colon, feces, and cloaca) from 20 juvenile ostriches, using 16S rRNA Illumina MiSeq sequencing. We found that direct PCR was highly comparable over a range of measures to the DNA extraction method in cecal, colon, and fecal samples. However, the two methods significantly differed in samples with comparably low bacterial biomass: cloacal and especially ileal samples. We also sequenced 100 replicate sample pairs to evaluate repeatability during both extraction and PCR stages and found that both methods were highly consistent for cecal, colon, and fecal samples (*r*_s_ > 0.7) but had low repeatability for cloacal (*r*_s_ = 0.39) and ileal (*r*_s_ = −0.24) samples. This study indicates that direct PCR provides a fast, cheap, and reliable alternative to conventional DNA extraction methods for retrieving 16S rRNA data, which can aid future gut microbiome studies.

**IMPORTANCE** The microbial communities of animals can have large impacts on their hosts, and the number of studies using high-throughput sequencing to measure gut microbiomes is rapidly increasing. However, the library preparation procedure in microbiome research is both costly and time-consuming, especially for large numbers of samples. We investigated a cheaper and faster direct PCR method designed to bypass the DNA isolation steps during 16S rRNA library preparation and compared it with a standard DNA extraction method. We used both techniques on five different gut sample types collected from 20 juvenile ostriches and sequenced samples with Illumina MiSeq. The methods were highly comparable and highly repeatable in three sample types with high microbial biomass (cecum, colon, and feces), but larger differences and low repeatability were found in the microbiomes obtained from the ileum and cloaca. These results will help microbiome researchers assess library preparation procedures and plan their studies accordingly.

## Abstract (precis)

The microbial communities of animals can have large impacts on their hosts, and the number of studies using high-throughput sequencing to measure gut microbiomes is rapidly increasing. However, the library preparation procedure in microbiome research is both costly and time-consuming, especially for large numbers of samples. We investigated a cheaper and faster direct PCR method designed to bypass the DNA isolation steps during 16S rRNA library preparation and compared it with a standard DNA extraction method. We used both techniques on five different gut sample types collected from 20 juvenile ostriches and sequenced samples with Illumina MiSeq. The methods were highly comparable and highly repeatable in three sample types with high microbial biomass (cecum, colon, and feces), but larger differences and low repeatability were found in the microbiomes obtained from the ileum and cloaca. These results will help microbiome researchers assess library preparation procedures and plan their studies accordingly.

## INTRODUCTION

It is becoming increasingly evident that the microbes animals harbor play an important role in regulating the physiology and behavior of individuals (1). For example, the human gut contains trillions of bacteria (2) that, together with other microorganisms, have large effects on human health and disease (3–5). Although the varied and prominent effects of the gut microbiome on hosts have been brought to the forefront by studies on humans, research on nonhuman organisms is rapidly expanding and illustrating the importance of host microbiomes for a range of ecological and evolutionary processes (6–12). This increase has been greatly aided by progress in sequencing technologies and genetic markers, such as 16S rRNA, that allow large numbers of bacterial communities to be characterized. However, the scope of microbiome studies continues to be limited by time-consuming laboratory procedures, in particular the isolation of DNA and the generation of amplicon libraries. As phenotypic variation is widespread in natural populations, the success of ecological and evolutionary studies on host microbiomes relies on large sample sizes, and so it is important to find fast, cost-effective, and reliable ways of processing microbiome samples.

The conventional way of generating amplicon libraries for microbiome studies is to first extract and purify DNA, for example, with kits such as the Mo Bio PowerSoil DNA isolation kit. This procedure is recommended by the Earth Microbiome Project (13, 14) and is widely used in human and nonhuman animal microbiota studies. The DNA extraction protocol involves mechanical and chemical lysis of cells and a DNA purification procedure that adds up to 32 separate steps (Table 1). One potentially faster and cheaper technique to prepare amplicon libraries is the recently developed direct PCR method. This method minimizes the DNA isolation steps because DNA is simply extracted in a buffer with a 10-min 95°C treatment prior to PCR amplification. To our knowledge, the accuracy of direct PCR versus DNA extraction for 16S rRNA sequencing of microbiomes has only been assessed once before, in human samples from four individuals and with the discontinued 454 pyrosequencing technique (15). The results of that comparison suggested that the direct PCR method was a highly viable alternative to the DNA extraction method (15). This information raises the question of whether direct PCR provides a cheaper and faster alternative to the conventional methods currently being used in nonhuman animal microbiome studies.

**TABLE 1:** Comparison of practical factors of the DNA extraction and direct PCR methods for gut microbiome studies

Method	Extraction time^c^	No. of protocol steps	Kit cost/sample (US$)	Storage of extracted DNA
DNA extraction^a^	~8 h (1–2 days)	32	6.90^d^	Long-term
Direct PCR^b^	45 min	3	2.30^e^	Short-term

a Information is shown for DNA extraction with the PowerSoil-htp 96-Well Soil DNA kit, 384 extractions (Mo Bio Laboratories, Inc.).

b Information is shown for direct PCR with the Extract-N-Amp Plant PCR kit, 100 extractions (Sigma-Aldrich).

c Time is estimated for parallel extraction of 96 to 192 samples.

d Price includes PCR reagents (Phusion High-Fidelity PCR master mix with HF buffer [Thermo Scientific]) that are not part of the DNA extraction kit.

e Price includes PCR reagents that are part of the kit.

We therefore evaluated microbial communities obtained with the direct PCR approach (a modification of the method of Flores et al. [15]) compared to the conventional DNA extraction technique (Mo Bio PowerSoil DNA isolation kit) using 16S rRNA Illumina MiSeq sequencing. We examined the performance of these two techniques across the length of the gut (ileum, cecum, and colon) and in two sample types commonly used for animal microbiome studies (feces and cloacal swabs) of juvenile ostriches. We used this species, age, and set of sample types as they are known to differ markedly in microbial composition (16) and microbial environment (17), enabling the assessment of both methods across different conditions and microbial communities. Furthermore, ostriches are very distant ecologically and phylogenetically from humans, which were used in the previous microbiome study evaluating the direct PCR method (15), and so if corresponding results between methods were found in both of these species, it would suggest that they may be applicable over a wide range of animal species. Our aims were to test if the direct PCR and DNA extraction methods yield similar results in terms of (i) community diversity and distance, (ii) specific bacterial taxa and abundances, and (iii) repeatability of replicates from the same samples.

## RESULTS

### Practical aspects of direct PCR and DNA extraction.

The total time spent extracting DNA with the direct PCR method was considerably shorter (45 min) than that required for the conventional DNA extraction method (8 h) (Table 1). The cost of using the direct PCR method was also lower, as was the number of steps in the protocol (Table 1). Nevertheless, the number of sequence reads obtained per sample (mean, 10,689 ± 2,745) did not differ between the DNA extraction method and the direct PCR method (two-sample *t* test: *t* = 1.25, df = 290.7, *P* = 0.21) (see Fig. S2 in the supplemental material).

10.1128/mSystems.00132-17.1 FIG S1  Illustrative overview of the experimental setup of gut samples. Download FIG S1, PDF file, 0.1 MB. Copyright © 2017 Videvall et al. 2017 Videvall et al. This content is distributed under the terms of the Creative Commons Attribution 4.0 International license.

10.1128/mSystems.00132-17.2 FIG S2  Number of reads per sample type and method. Download FIG S2, PDF file, 0.2 MB. Copyright © 2017 Videvall et al. 2017 Videvall et al. This content is distributed under the terms of the Creative Commons Attribution 4.0 International license.

### Description of the microbiomes obtained with direct PCR and DNA extraction.

**FIG 1:**
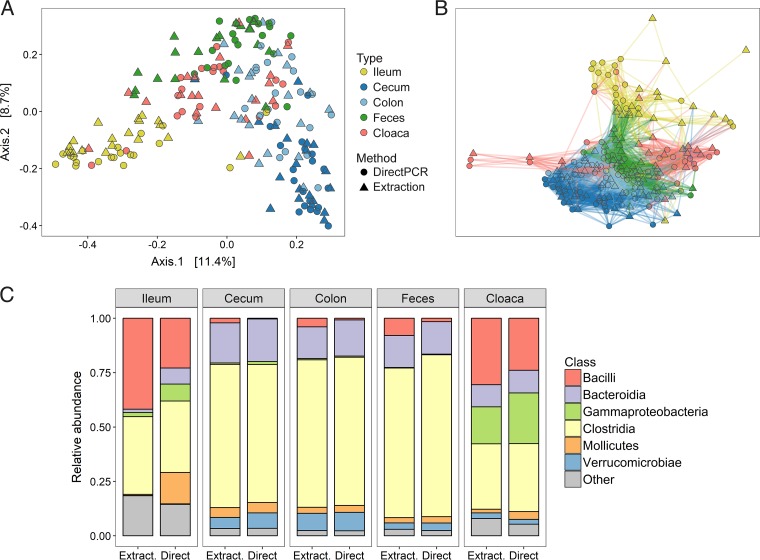
Microbiomes of different gut samples obtained with the direct PCR and conventional DNA extraction methods. (A, B) PCoA (A) and network analysis (B) of all sample types from all individuals prepared with the two methods. (C) Relative abundances of bacterial classes in the different sample types displayed for the two methods. Extract., DNA extraction method; Direct, direct PCR method.

Samples clustered strongly according to sample type in both principal-coordinate analysis (PCoA) (Fig. 1A) and network analysis (Fig. 1B), although some minor separation between the direct PCR and DNA extraction methods was evident. The two library preparation methods yielded fairly consistent patterns for the total number of operational taxonomic units (OTUs) per bacterial class and sample type but also reflected some notable differences in taxon composition (Fig. 1C). Specifically, *Bacilli* were slightly more abundant in all sample types with the DNA extraction method relative to the direct PCR method, and the ileum in particular showed the largest class differences, with a higher abundance of *Mollicutes*, *Gammaproteobacteria*, and *Bacteroidia* with the direct PCR method (Fig. 1C).

**FIG 2:**
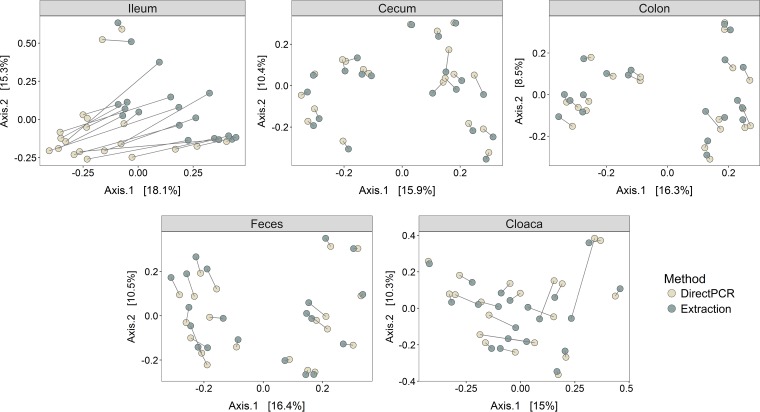
PCoAs of Bray-Curtis distances of different sample types. Lines between points denote identical samples prepared with the direct PCR (light dots) and DNA extraction (dark dots) methods. Values in brackets are the percent variances explained by the PCoAs.

To further investigate if the microbiota of samples from the direct PCR and DNA extraction methods differed depending on the gut site, we performed separate PCoAs for the five sample types. The cecum, colon, and feces showed very high correspondence in beta diversity for identical samples prepared with direct PCR and DNA extraction, as they clustered by individual and not method, whereas differences were much greater for cloacal and, in particular, ileal samples (Fig. 2).

### Differences in microbiomes obtained with direct PCR and DNA extraction.

**FIG 3:**
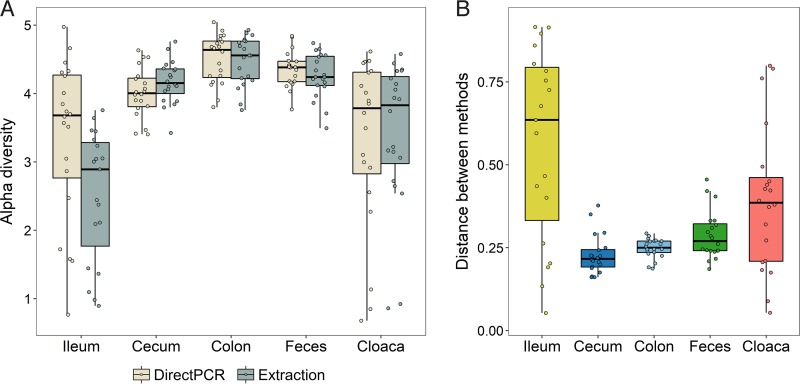
Differences in the microbiomes obtained with the direct PCR and DNA extraction methods. (A) Alpha diversity (Shannon index) of all samples within the different sample types for the direct PCR and DNA extraction methods. (B) Bray-Curtis distances between identical samples prepared with the direct PCR and DNA extraction methods.

Next we evaluated differences in alpha diversity (OTU richness) between the direct PCR and DNA extraction methods for the different sample types (Fig. 3A). There were no differences in alpha diversity between the two methods in the colon, feces, and cloaca (paired Wilcoxon signed-rank test: *n*_pairs_ = 20/type, *P* > 0.4) (Fig. 3A). However, alpha diversity was significantly higher in direct PCR samples than in DNA extraction samples for the ileum (*n*_pairs_ = 19, *V* = 5, *P* < 0.0001) and significantly lower in direct PCR cecal samples (*n*_pairs_ = 20, *V* = 199, *P* = 0.0001) (Fig. 3A). Correlation analyses of alpha diversity between the two methods also showed higher diversity for ileal direct PCR samples and slightly lower diversity for cecal direct PCR samples, but the strength of the correlations between methods was generally high for all sample types (*r* = 0.56 to 0.91) (Fig. S3).

10.1128/mSystems.00132-17.3 FIG S3  Correlation of alpha diversity (Shannon index) between the DNA extraction and direct PCR methods separately for each sample type. *r* values are Pearson correlation coefficients. Download FIG S3, PDF file, 0.5 MB. Copyright © 2017 Videvall et al. 2017 Videvall et al. This content is distributed under the terms of the Creative Commons Attribution 4.0 International license.

Dissimilarities in the microbiome composition between samples, as calculated by the Bray-Curtis distance measure, showed significant effects of method, sample type, individual, and the interaction between method and sample type (permutational multivariate analysis of variance [PERMANOVA]: all effects, *P* < 0.001). The overall variance explained by method and by method interacting with sample type was, however, extremely small (*R*^2^ = 0.014 and 0.019, respectively), whereas the variances explained by host individual (*R*^2^ = 0.283) and sample type (*R*^2^ = 0.201) were substantially larger.

Examination of the Bray-Curtis distances between the two methods within sample types revealed relatively short distances for the cecum, colon, and feces (means, 0.23, 0.25, and 0.29, respectively), while the cloaca (mean, 0.39) and, most notably, the ileum (mean, 0.56) displayed much greater distances and much higher variances (Fig. 3B). Specifically, the distances between identical samples from the ileum prepared with each method were significantly higher than the corresponding distances between identical samples from the cecum, colon, and feces (*n*_pairs_ = 19, *V* > = 165, *q* < 0.009 for each of these three tests) (all pairwise comparisons between sample types [10 tests] tested with the paired Wilcoxon signed-rank test with *P* values corrected for multiple testing). Cloacal samples showed significantly greater distances than cecal (*n*_pairs_ = 20, *V* = 21, *q* = 0.005) and colon (*n*_pairs_ = 20, *V* = 34, *q* = 0.013) samples, and finally, cecal samples showed slightly shorter distances than fecal samples (*n*_pairs_ = 20, *V* = 40, *q* = 0.023) (Fig. 3B).

### Differences in OTU abundance with direct PCR and DNA extraction.

Calculation of the correlation between the average OTU abundances of the direct PCR and extraction methods revealed very high correlation coefficients for the cecum, colon, and feces (*r*_s_ = 0.84 to 0.86), weaker for the cloaca (*r*_s_ = 0.60), and negative for the ileal samples (*r*_s_ = −0.17) (Fig. S4).

10.1128/mSystems.00132-17.4 FIG S4  Correlation of OTU abundances between the DNA extraction and direct PCR methods separately for each sample type. Abundances have been normalized in accordance with the DESeq2 method and log transformed (+0.1) for graphical purposes. *r* values are Spearman rank correlation coefficients. Download FIG S4, PDF file, 1.1 MB. Copyright © 2017 Videvall et al. 2017 Videvall et al. This content is distributed under the terms of the Creative Commons Attribution 4.0 International license.

**FIG 4:**
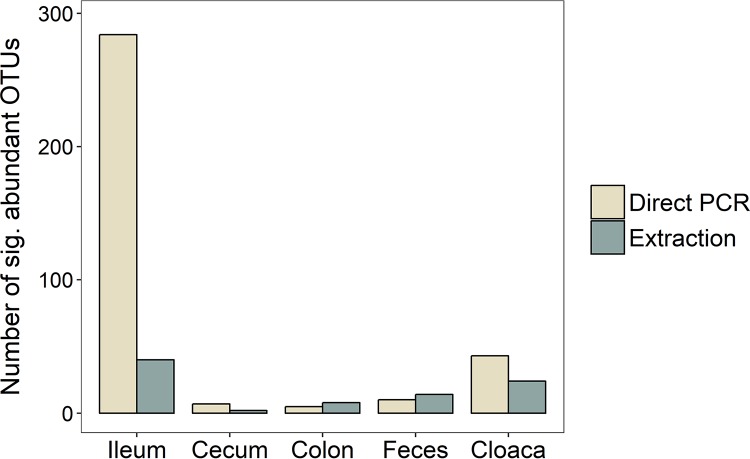
Numbers of OTUs with significantly higher abundance in either library preparation method for the different sample types.

Analyses of differences in the abundance of specific OTUs obtained by the two methods resulted in very few significantly different OTUs in the cecum (*n* = 9), colon (*n* = 13), and feces (*n* = 24) (Fig. 4). However, there were many more in the cloaca (*n* = 67), and the ileum demonstrated a staggering 324 OTUs that differed significantly between the DNA extraction and direct PCR methods (Fig. 4). Notably, the vast majority of significantly different OTUs across all sample types (80%) had higher abundances when direct PCR was used than when DNA extraction was used.

**FIG 5:**
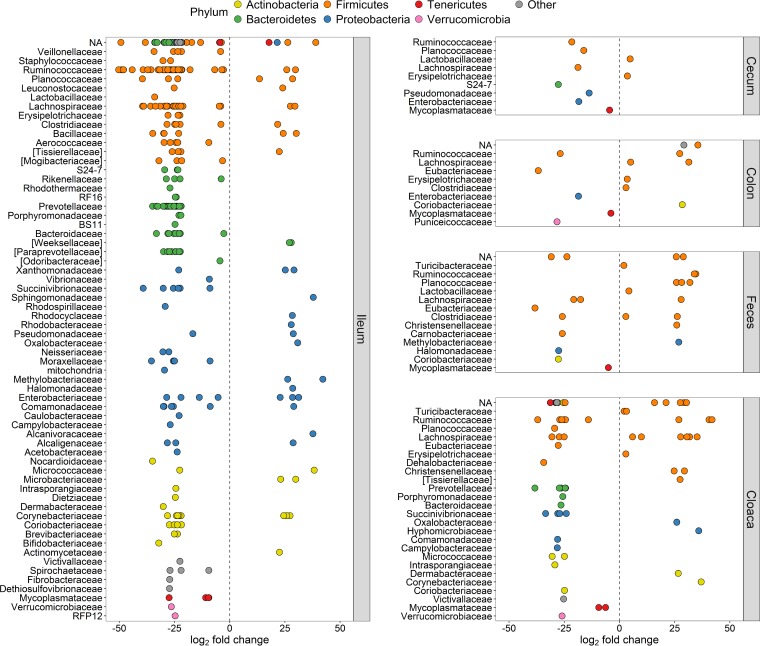
Significantly differentially abundant OTUs (*q* < 0.01) between the direct PCR and conventional DNA extraction methods. *y* axes show taxonomic families, and all OTUs have been colored within their respective phylum and separated by sample type. Positive log_2_ fold changes indicate higher relative OTU abundance in the DNA extraction method, and negative log_2_ fold changes signify higher abundance in the direct PCR method. NA, OTUs without family classification.

Comparison of the exact OTUs that had significantly different abundances in the five sample types showed that they were unique to each sample type (i.e., OTUs were only significantly different within one type) (Table S2). However, we found one genus, *Mycoplasma*, with significantly different OTUs present in all sample types. All significantly different *Mycoplasma* OTUs (family *Mycoplasmataceae*) had higher relative abundances in the samples from the direct PCR method than from the DNA extraction method (Fig. 5). Other genera with significantly different abundances in multiple sample types were, e.g., *Anaerofustis* (higher abundance with the direct PCR method in colon, fecal, and cloacal samples) and *Klebsiella* (more numerous with the direct PCR method in ileum, cecum, and colon samples). The genus *Prevotella* (class *Bacteroidia*) was the most prevalent in the list of significantly different genera, representing 43 unique OTUs in the cloaca and ileum, all (100%) of which had higher abundance in the direct PCR samples (Table S2).

The phylum with the highest number of significantly differentially abundant OTUs was *Firmicutes* (*n* = 210 in total), and in particular the class *Clostridia* (*n* = 171 in total) (Fig. 5; Table S2), which was in majority in most sample types (Fig. 1C). The genera that comprised the most significant differentially abundant OTUs between the two methods were *Oscillospira* (class *Clostridia*), *Mycoplasma* (class *Mollicutes*), and *Coprococcus* (class *Clostridia*) (Table S2).

### Repeatability of replicate samples with direct PCR and DNA extraction.

**FIG 6:**
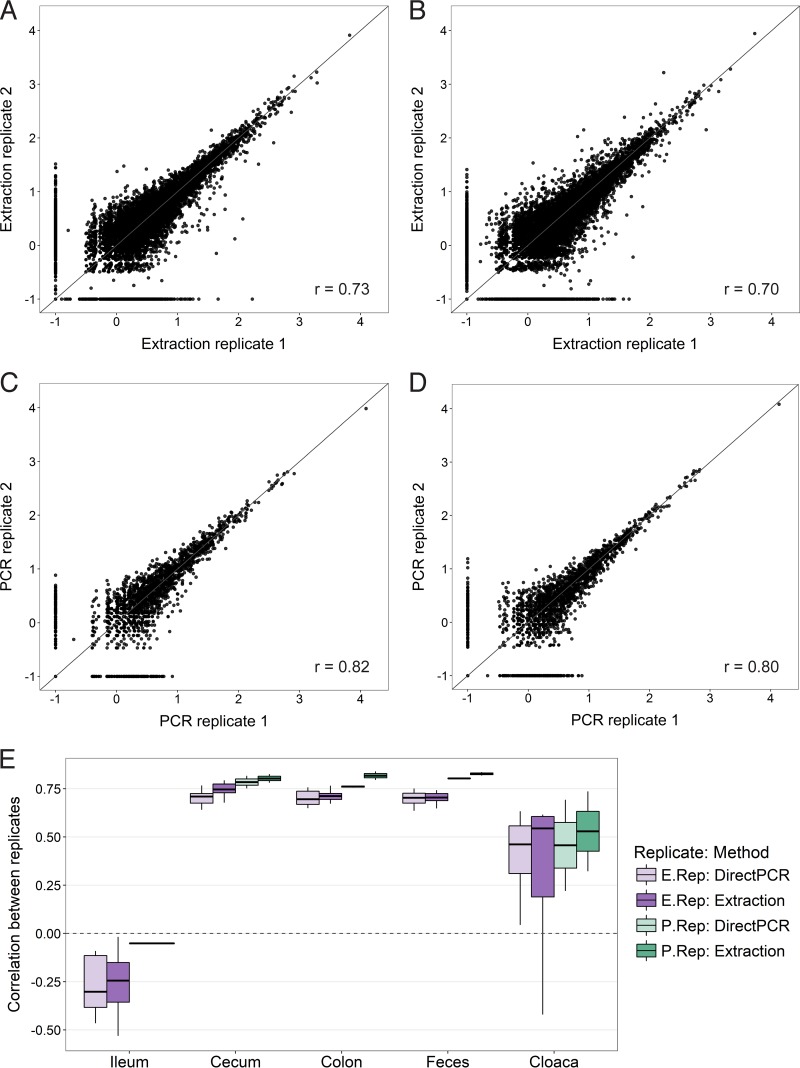
Repeatability of OTU abundances between replicate samples. Scatterplots show abundances of all OTUs in extraction replicates obtained with the DNA extraction method (A), extraction replicates obtained with the direct PCR method (B), PCR replicates obtained with the DNA extraction method (C), and PCR replicates obtained with the direct PCR method (D). OTU abundances have been normalized in accordance with the DESeq2 method and log transformed (+0.1) for graphical purposes. *r* values are Spearman rank correlation coefficients. (E) Box plot of correlation coefficients (Spearman rank correlation) of OTU abundances calculated separately for all 100 replicate sample pairs.

Next, we evaluated the repeatability of the DNA extraction and direct PCR methods by calculating correlations of OTU abundances and diversity between pairs of replicate samples. For the “extraction replicates,” the correlation coefficient of OTU abundance was almost identical for the DNA extraction method (*r*_s_ = 0.73; Fig. 6A) and the direct PCR method (*r*_s_ = 0.70; Fig. 6B). For the “PCR replicates,” the strength of the correlation was slightly greater but again similar for the two methods (DNA extraction, *r*_s_ = 0.82, Fig. 6C; direct PCR, *r*_s_ = 0.80, Fig. 6D).

When we partitioned the OTU abundance data by sample type, we observed large differences in repeatability (Fig. 6E). The cecal, colon, and fecal samples had the strongest correlations between replicates, with both methods having an average *r*_s_ of 0.70 to 0.74 for the extraction replicates and an average *r*_s_ of 0.76 to 0.83 for the PCR replicates (Fig. 6E). In contrast, the extraction replicates from the cloaca were characterized by a much weaker mean correlation (*r*_s_ = 0.36), as were the cloacal PCR replicates (*r*_s_ = 0.49), and the correlations between ileal replicates were even negative (extraction replicates, *r*_s_ = −0.27; PCR replicates, *r*_s_ = −0.05) (Fig. 6E).

Finally, we examined the correlation between alpha diversity estimates in the replicate samples to evaluate the repeatability of the community between methods. Relative to the OTU abundance data, there was higher repeatability in alpha diversity for cloacal and ileal samples (*r* = 0.79 to 0.96), while the cecal, colon, and fecal samples again had high repeatability for both methods (*r* = 0.70 to 0.97) (Fig. S5 and S6).

## DISCUSSION

This study shows that the direct PCR method provides results highly comparable to those of the widely used and recommended DNA extraction method in the analyses of ostrich gut microbiomes. Both techniques gave qualitatively and quantitatively similar estimates of microbial diversity and abundance in cecal, colon, and fecal samples and were highly repeatable for these sample types. However, the two methods present dissimilar microbiomes in cloacal and, in particular, ileal samples, recovering large differences and poor repeatability in OTU abundance across replicates. We discuss hypotheses that may explain why these methods perform well with some sample types but not others.

During amplicon library preparation, PCR products from the ileal and cloacal samples were much weaker than those from the other sample types, presumably because of the lower microbial biomass in these samples. A low initial template DNA concentration may reduce repeatability in two ways. First, low DNA concentrations will introduce stochasticity in what bacterial species in the original samples are amplified. Second, small differences between the direct PCR and DNA extraction methods can become exaggerated in low-DNA samples because the sequence coverage will be higher for low-abundance OTUs as a result of equal quantities of PCR product from all samples being used before sequencing. However, the uniform direction observed between identical samples in the PCoA of ileum samples (Fig. 2) indicated that there were consistent differences between the two methods. This result was also supported by the correspondence in alpha diversity of cloacal and ileal samples both between replicates (Fig. S5 and S6) and between the methods (Fig. S3), suggesting that although it is difficult to measure the relative abundances of specific bacterial taxa when DNA concentrations are low, it may still be possible to accurately measure the community composition with both of these methods.

The direct PCR samples from the ileum had significantly higher alpha diversity (Fig. 3A) and higher relative abundances of the majority of differentially abundant OTUs (Fig. 4) than those obtained with the DNA extraction method. One potential reason for this difference is that more DNA is lost during the DNA extraction procedure, which is column based with several wash and transfer steps. This method is known to be associated with high DNA loss, whereas with direct PCR, the individual samples are contained in just one plate well during the full extraction procedure. In samples with low starting DNA concentrations, direct PCR may therefore be superior to conventional extraction methods at recovering rare bacterial taxa. The higher diversity in the ileal direct PCR samples could also be a consequence of the regular *Taq* polymerase included in the direct PCR mixture, which is slightly more error prone than the Phusion High-Fidelity polymerase used for amplification in the DNA extraction protocol. A higher error rate during the direct PCR amplification step could potentially yield spurious OTUs and raise the alpha diversity and the number of differentially abundant OTUs. However, *Taq* polymerase does not explain the consistent changes observed in the PCoA (Fig. 2), the correlation in diversity between the methods (Fig. S3), or the changes in the relative abundance of specific taxonomic groups (Fig. 1C and 5).

Despite high correspondence of abundance estimates across most OTUs, there were some consistent differences in the abundance of specific taxa between the direct PCR and DNA extraction methods. It is possible that morphological differences between bacterial groups may influence the efficiency with which the two methods recover DNA. For example, compared to the DNA extraction method, we found that direct PCR had higher relative abundances of *Mollicutes* (small bacteria without a cell wall) and fewer members of the class *Bacilli* (Gram-positive bacteria with a thick cell wall) in ileal samples. The direct PCR method uses chemical and heat shock treatments to lyse cells, whereas the DNA extraction method lyses cells with mild heat, chemical, and mechanical treatments. This difference could, in theory, have different effects on the release of DNA, according to the bacterial cell wall structure. The direct PCR approach previously tested on human samples (15) found members of the family *Prevotellaceae* in higher abundance in several tongue samples and members of the family *Veillonellaceae* in higher abundance in gut samples. We obtained similar results, with 43 significantly different OTUs belonging to the family *Prevotellaceae* and 5 significantly different *Veillonellaceae* OTUs, all more abundant in ileal and cloacal samples with the direct PCR method (Fig. 5), suggesting that direct PCR may be better at recovering these specific taxa. Furthermore, we found *Mycoplasma* spp. present in significantly higher abundance in all sample types with the direct PCR method (Table S2). *Mycoplasma* spp. are sometimes present as laboratory contaminants (18); however, sequencing of negative controls of the direct PCR kits produced practically zero occurrences of *Mycoplasma* spp. (0 to 2 reads) and extremely few sequences of other bacteria (66 to 193 reads), proving contamination by the direct PCR kits highly unlikely.

With these results, there is now evidence that the direct PCR kit works well with various microbiome sample types in two different animal species: human skin, tongue, and fecal samples (15) and different ostrich gut sections (this study). Ostriches are predominantly herbivores, and their gut microbiota as juveniles is dominated by *Firmicutes*, followed by *Bacteroidetes* (Fig. 1C). This taxon composition is similar to that of chickens and turkeys of the same age (19, 20), to adults of the same species (E. Videvall et al., unpublished data), and to herbivorous lizards (21), pigs (22), ground squirrels (7), and many other mammalian (23, 24) and nonmammalian (25) vertebrates. Despite overall similarities in the composition of dominant phyla, it remains to be established if direct PCR performs as well in other animal species. Nevertheless, given the large ecological and phylogenetic distances between humans and ostriches and the different sample types evaluated so far, it appears very promising.

In summary, the direct PCR and conventional DNA extraction methods gave highly similar estimates of community composition and overall OTU abundance in gut microbiomes from high-microbial-biomass samples, such as those from the cecum, colon, and feces. The direct PCR protocol is, however, cheaper, takes less time, and involves 29 fewer laboratory steps, thereby reducing the risk of contamination and human error (Table 1). These practical advantages, combined with the fact that direct PCR is possibly more sensitive at measuring diversity in samples with low DNA concentrations, highlight direct PCR as an excellent alternative to the DNA extraction method. We hope that these results will aid researchers in the planning of gut microbiome studies and stimulate further research into reliable and efficient methods.

## MATERIALS AND METHODS

### Sample collection.

The sample collection has been outlined in detail by Videvall et al. (16). Briefly, samples were collected from juvenile ostriches (*Struthio camelus*) kept at the Western Cape Department of Agriculture's ostrich research facility in Oudtshoorn, South Africa. Samples were collected from 20 randomly selected birds (10 individuals four weeks old and 10 individuals six weeks old). Five different sample types were collected from each individual: feces, cloacal swabs, and gut contents from the ileum, cecum, and colon, together with controls for the cloacal swabs (16). All procedures were approved by the Departmental Ethics Committee for Research on Animals (DECRA) of the Western Cape Department of Agriculture, reference no. R13/90. The samples were collected between 28 October and 12 November 2014 in 2-ml plastic microtubes (Sarstedt catalog no. 72.693) and stored at −20°C.

### Sample preparation.

All sample handling, DNA isolation, and library preparations were performed inside a UV hood to minimize contamination. Before starting the work, boxes with sterile plastic consumables and containers with reagents were UV sterilized for at least 1 h in the closed hood. DNA extraction, library preparation, and sequencing took place between 5 October and 13 November 2015. Sample slurries were prepared from the five different sample types with guidance from Flores et al. (15). Ileal, cecal, colon, and fecal samples were thawed, kept on ice, and vortexed vigorously. From the ileal and cecal samples, 50 to 100 µl was taken with a pipette, and from the colon and fecal samples, the tip of a sterile disposable spatula (approximately 20 to 80 mg) was used. The samples were dissolved in 2 ml of nuclease-free water (Ambion catalog no. AM9938) in 50-ml sterile tubes. For cloacal and control swab samples, 250 μl of sterile water was added directly to the original tube containing the swab.

The tubes were vigorously vortexed for 30 s, and slurries (1 ml of gut and fecal slurries and 100 μl of swab slurries) were immediately transferred to presterilized X30 Deep 96-well plates (Axygen catalog no. P-DW-20-C-S-IND) and kept on ice. Plates were sealed with ImpermaMat X50 chemical-resistant sealing mats (Axygen catalog no. AM-2ML-RD-IMP) and kept at −25°C until DNA isolation. Each sample slurry was used in both the subsequent DNA extraction and direct PCR methods, creating two identical sample sets for comparison of the two preparation methods.

### DNA isolation and 16S rRNA library preparation using the DNA extraction method.

We used the PowerSoil-htp 96-well soil DNA isolation kit (Mo Bio Laboratories catalog no. 12955-4). DNA extraction with this kit includes mechanical and chemical lysis of cells and subsequent column-based DNA purification in a process with 32 discrete steps that takes around 8 h to perform for 96 samples. The DNA isolation procedure was performed in accordance with the manufacturer’s protocol, with slight modifications (15) (Earth Microbiome Project DNA extraction protocol, version 4_13, http://www.earthmicrobiome.org/emp-standard-protocols/16s/), as detailed below. Sample slurry plates were thawed and shaken for 90 s at 20 Hz in a TissueLyser (Qiagen) to mix them and then centrifuged (up to 200 × *g*) in a plate centrifuge. A volume of 25 μl of slurry was pipetted from the slurry plates to Mo Bio PowerSoil-htp Bead Plates, 60 μl of solution C1 was added, and the plates were sealed and incubated for 10 min at 65°C before the TissueLyser step in the manufacturer’s protocol, which was followed thereafter. DNA was eluted in 100 μl of solution C6.

Amplicon libraries (one for each sample) targeting the V3 and V4 regions of the 16S small-subunit rRNA gene were prepared for sequencing according to the Illumina 16S Metagenomic Sequencing Library Preparation Guide (part number 15044223, revision B), with slight modifications (detailed below). Each PCR mixture (25 µl) contained 10 μl of DNA extract, each Illumina fusion primer at 0.5 µM with the gene-specific forward and reverse primers Bakt_341F and Bakt_805R (26), and 1× Phusion High-Fidelity PCR master mix with HF buffer (Thermo Scientific catalog no. F-531S). The cycling conditions were 98°C for 30 s, followed by 25 cycles of 98°C for 10 s, 56°C for 15 s, and 72°C for 20 s, and a final extension step of 72°C for 10 min. The amplicons were purified with AmPure XP beads (Agencourt catalog no. A63881) in a ratio of 1:0.8 (PCR product to bead solution) and freshly prepared 80% ethanol in accordance with the manufacturer’s protocol.

The purified amplicons were each dissolved in 43 μl of nuclease-free water (Ambion catalog no. AM9938), and 15 μl was transferred to a new PCR plate for subsequent dual-index PCR with Nextera Index kit V2 set C or D (Illumina catalog no. FC-131-2003 and FC-131-2004) to individually label the amplicons. The index PCRs (50 μl) contained 5 μl each of forward and reverse Nextera Index primers and 1× Phusion High-Fidelity PCR master mix with HF buffer (Thermo Scientific catalog no. F-531S). The cycling conditions were 98°C for 30 s followed by eight cycles of 98°C for 10 s, 62°C for 30 s, and 72°C for 30 s, and a final extension step of 72°C for 10 min.

The individually indexed amplicons were purified with AmPure XP beads (Agencourt catalog no. A63881) in a ratio of 1:1.12 (PCR product to bead solution) and freshly prepared 80% ethanol in accordance with the manufacturer’s protocol. The purified individually indexed amplicons were then each dissolved in 43 μl of nuclease-free water (Ambion catalog no. AM9938), and 38 μl was transferred to a new PCR plate. Amplicons were quantified on the FLUOstar Omega plate reader system (BMG Labtech) with the Quant-iT PicoGreen dsDNA kit (Invitrogen catalog no. P7589). Weak PCR products (<4.5 ng/μl) were evaporated to increase the DNA concentration and then requantified. All amplicons were pooled in equimolar amounts and analyzed on a 2100 Bioanalyzer (Agilent Technologies catalog no. G2940CA) before sequencing.

### 16S rRNA library preparation using the direct PCR method.

The direct PCR approach was performed with the Extract-N-Amp Plant PCR kit (Sigma-Aldrich catalog no. XNAP2) with guidance from Flores et al. (15). In this method, 96 samples are prepared without DNA purification for PCR amplification in three steps as detailed below. Sample slurry plates were thawed and shaken for 90 s at 20 Hz in a TissueLyser (Qiagen) to mix and then centrifuged (up to 200 × *g*) in a plate centrifuge. A volume of 25 μl of slurry was pipetted from each sample in the slurry plates into 100 μl of Extract-N-Amp Plant PCR kit extraction buffer in presterilized X30 Deep 96-well plates (Axygen catalog no. P-DW-20-C-S-IND 96). The plates were sealed with X50 ImpermaMat chemical-resistant sealing mats (Axygen catalog no. AM-2ML-RD-IMP). The plates were heated in a water bath for 10 min at 95°C and then centrifuged at 2,500 × *g* for 5 min before the addition of 100 μl of Extract-N-Amp Plant PCR kit Dilution buffer to each well by gentle pipetting. The plates were kept at −25°C until direct PCR.

Amplicon libraries were created for the resulting direct PCR extracts. The same Illumina adaptor-modified forward primer Bakt_341F and reverse primer Bakt_805R (26) as the DNA extraction method samples were used. Each PCR mixture (25 µl) contained 5 μl of Extract-N-Amp DNA extract, each Illumina fusion primer at 0.5 µM, and 1× Extract-N-Amp PCR Ready Mix (Sigma-Aldrich catalog no. XNAP2). The cycling conditions were 94°C for 3 min, followed by 25 cycles of 94°C for 30 s, 50°C for 30 s, and 72°C for 45 s, and a final extension step of 72°C for 10 min.

The amplicons were purified, individually labeled, quantified, and pooled for sequencing using the same method as described above for the samples prepared with the DNA extraction method. The direct PCR samples were labeled with Nextera Index kit V2 set A or B (Illumina catalog no. FC-131-2001 and FC-131-2002).

### Replicate samples.

We created two sets of replicate samples for each preparation method to evaluate repeatability (Fig. S1). The first set, extraction replicates, included 80 sample pairs (*n* = 40 pairs for each method, partitioned into 8 pairs per sample type) and was created before the DNA extraction and direct PCR procedures by dispensing the slurry from the same sample into two wells situated on two different plates. The second set, PCR replicates, was created by amplifying from the same DNA extract in two separate PCR wells. The PCR replicates included a total of 20 pairs of samples (*n* = 10 pairs for each method, partitioned into 2 pairs per sample type). These two replicate levels were created to evaluate at what stage in the library preparation procedure we could detect potential differences, if repeatability differed.

### Amplicon sequencing.

DNA sequencing was performed in accordance with the Illumina 16S Metagenomic Sequencing Library Preparation Guide (part number 15044223, revision B) at the DNA Sequencing Facility, Department of Biology, Lund University. The equimolarly pooled amplicon libraries and 10% PhiX spike-in with the PhiX Control kit V3 (Illumina catalog no. FC-110-3001) were sequenced in one 300-bp paired end run on an Illumina MiSeq platform with the MiSeq Reagent kit V3 (600 cycles) (Illumina catalog no. MS-102-3003). The pool of amplicon libraries (and PhiX) was added as 8 pM and produced at a cluster density of 891,000/mm^2^. To summarize, a total of 321 different amplicon libraries were part of this study. The amplicon libraries represented 100 unique ostrich gut microbiome samples (*n* = 20 per sample type), 2 control and 2 blank samples, 40 extraction replicates, and 10 PCR replicates that were all prepared with both the DNA extraction and direct PCR methods (Table S1; Fig. S1). An additional 11 control swabs and 2 blank samples from a subsequent run were also evaluated to increase the number of controls (Table S1).

10.1128/mSystems.00132-17.7 TABLE S1  Sample information. Download TABLE S1, XLS file, 0.1 MB. Copyright © 2017 Videvall et al. 2017 Videvall et al. This content is distributed under the terms of the Creative Commons Attribution 4.0 International license.

10.1128/mSystems.00132-17.8 TABLE S2  OTUs significantly differentially abundant between the DNA extraction and direct PCR methods. Download TABLE S2, TXT file, 0.2 MB. Copyright © 2017 Videvall et al. 2017 Videvall et al. This content is distributed under the terms of the Creative Commons Attribution 4.0 International license.

### Data processing.

The 16S rRNA amplicon sequences were quality controlled with FastQC (v. 0.11.5) (27) together with MultiQC (28). Primers were removed from the sequences with Trimmomatic (v. 0.35) (29), and the forward reads were retained for analyses. Quality filtering of the reads was executed with the script multiple_split_libraries_fastq.py from QIIME (v. 1.9.1) (30). All bases with a Phred score of <25 at the 3′ end of reads were trimmed, and samples were multiplexed into a single high-quality multi-fasta file.

OTUs were assigned and clustered with Deblur (v. 1.0.0) (31). Deblur circumvents the problems surrounding clustering of OTUs at an arbitrary threshold by obtaining single-nucleotide resolution OTUs (100% sequence identity approach) after correcting for Illumina sequencing errors. This results in exact sequence variants, also called amplicon sequence variants, oligotypes, zero-radius OTUs, and sub-OTUs. To avoid confusion, we chose to call these units OTUs, but it should be noted that they differ from the traditional 97% clustering approach, as they provide more accurate estimates (31–33). The minimum-reads option was set to 0 to disable filtering inside Deblur, and all sequences were trimmed to 220 bp. We used the biom table produced after both positive and negative filtering, which, by default, removes any reads that contain PhiX or adapter sequences and only retains sequences matching known 16S rRNA sequences. Additionally, PCR-originating chimeras were filtered from reads inside Deblur (31).

Taxonomic assignment of OTUs was performed using the Greengenes database (34). We filtered all samples on a minimum read count of 1,000 sequences, resulting in 6 of 321 samples being directly excluded (3 blank and 3 ileal samples). We further filtered all OTUs that only appeared in one sample, resulting in 4,290 OTUs remaining out of an initial 18,689. All samples with technical replicates (both extraction and PCR replicates) had the sequence data merged within their respective sample type (i.e., ileum.rep1 plus ileum.rep2) to increase the amount of sequence information per sample for all analyses except repeatability analyses, where the replicates were evaluated separately. We present results from nonrarefied data in this study, as recommended by McMurdie and Holmes (35).

### Data analyses.

Analyses were performed in R (v. 3.3.2) (36), and plots were made with phyloseq (37) and ggplot2 (38). We calculated the alpha diversity of samples with the Shannon measure by using the absolute abundance of reads and distance measures with the Bray-Curtis distance method on relative read abundances in phyloseq (v. 1.19.1) (37). The Bray-Curtis distance metric was used because we wanted to evaluate the ability of the two methods to recover identical OTUs (100% nucleotide identity) in similar abundances, as opposed to phylogenetic distance metrics (e.g., UniFrac), which risk showing strong correspondence between dissimilar OTUs that are phylogenetically close. Community level microbiome differences between the direct PCR and DNA extraction methods were examined with PERMANOVA on Bray-Curtis distances by using the Adonis function in vegan (v. 2.4-2) with 1,000 permutations (39). Sequencing of blank (negative) samples resulted in very few reads (mean, 1,431 ± 1,367), and most of the taxa are similar to contaminants described by Salter et al. (40). Control swabs (which were sampled to control for cloacal swabs) showed a microbial composition highly dissimilar from that of all other samples (analyzed in 16), and therefore we did not include these in any further analyses.

To evaluate bacterial abundances, we first filtered out all OTUs with fewer than 10 sequence reads and then, with DESeq2 (v. 1.14.1), counts were modeled with a local dispersion model and normalized per sample using the geometric mean (41). Differential abundances between the preparation methods were subsequently tested in DESeq2 with a negative binomial Wald test using individual identity as a factor and with the beta prior set to false (41). The results for specific comparisons were extracted (e.g., feces direct PCR versus feces extraction) and *P* values were corrected with the Benjamini and Hochberg false-discovery rate for multiple testing (42). OTUs were labeled significantly different if they had a corrected *P* value (*q* value) of <0.01.

We examined the repeatability of the two methods by evaluating the strength of the correlation in normalized OTU abundance between paired sample replicates. This approach was performed separately for the two methods and for the two replicate sets (extraction replicates and PCR replicates). Correlation coefficients were calculated using Spearman’s rank correlations on all OTUs with nonzero abundances.

### Data availability.

Sequence data obtained in this study have been uploaded to the European Nucleotide Archive under accession number PRJEB22648.

10.1128/mSystems.00132-17.5 FIG S5  Repeatability of alpha diversity (Shannon index) between extraction replicates from the DNA extraction method separately for each sample type. *r* values are Pearson correlation coefficients. Download FIG S5, PDF file, 0.4 MB. Copyright © 2017 Videvall et al. 2017 Videvall et al. This content is distributed under the terms of the Creative Commons Attribution 4.0 International license.

10.1128/mSystems.00132-17.6 FIG S6  Repeatability of alpha diversity (Shannon index) between extraction replicates from the direct PCR method separately for each sample type. *r* values are Pearson correlation coefficients. Download FIG S6, PDF file, 0.4 MB. Copyright © 2017 Videvall et al. 2017 Videvall et al. This content is distributed under the terms of the Creative Commons Attribution 4.0 International license.
