# Supplementary material for: Direct PCR Offers a Fast and Reliable Alternative to Conventional DNA Isolation Methods for Gut Microbiomes
Source: mSystems. 2017 Nov 21;2(6):e00132-17. doi: 10.1128/mSystems.00132-17 (PMC5698494; doi:10.1128/mSystems.00132-17)
Supplement: FIG S1 [file sys006172153sf3.pdf]

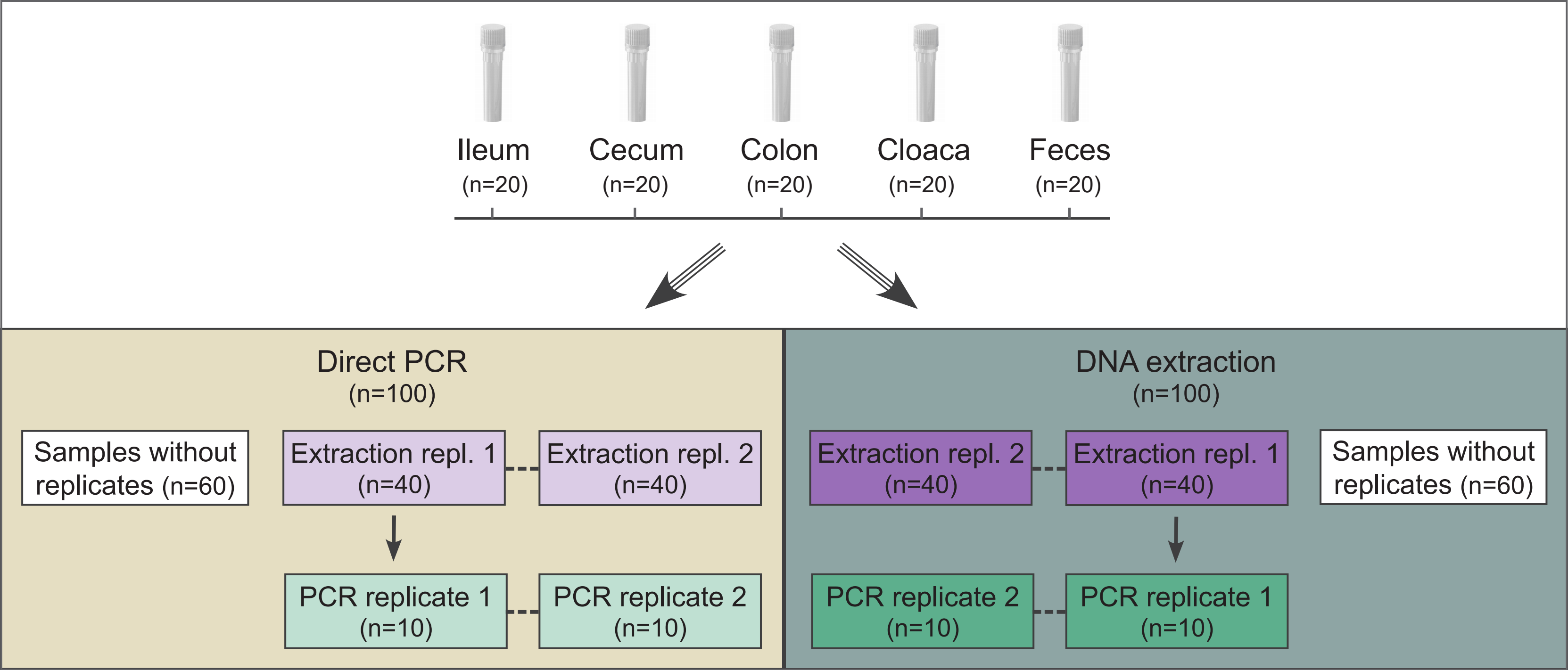

Ileum
(n=20)
Cecum
(n=20)
Colon
(n=20)
Cloaca
(n=20)
Feces
(n=20)
Direct PCR
(n=100)
Samples without replicates (n=60)
Extraction repl. 1
(n=40)
Extraction repl. 2
(n=40)
PCR replicate 1
(n=10)
PCR replicate 2
(n=10)
DNA extraction
(n=100)
Extraction repl. 2
(n=40)
Extraction repl. 1
(n=40)
Samples without replicates (n=60)
PCR replicate 2
(n=10)
PCR replicate 1
(n=10)
