# Supplementary figures and images for: Direct PCR Offers a Fast and Reliable Alternative to Conventional DNA Isolation Methods for Gut Microbiomes
Source: mSystems. 2017 Nov 21;2(6):e00132-17. doi: 10.1128/mSystems.00132-17 (PMC5698494; doi:10.1128/mSystems.00132-17)

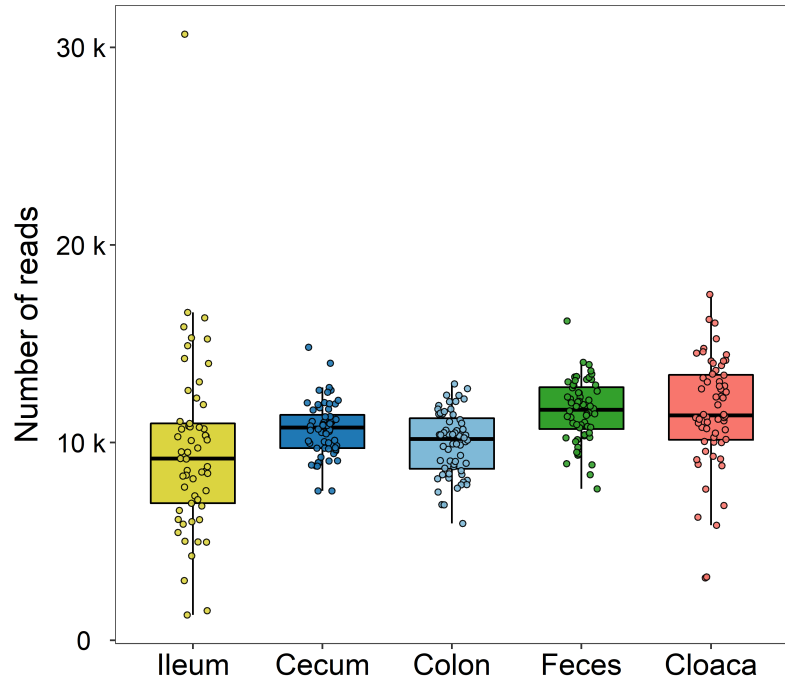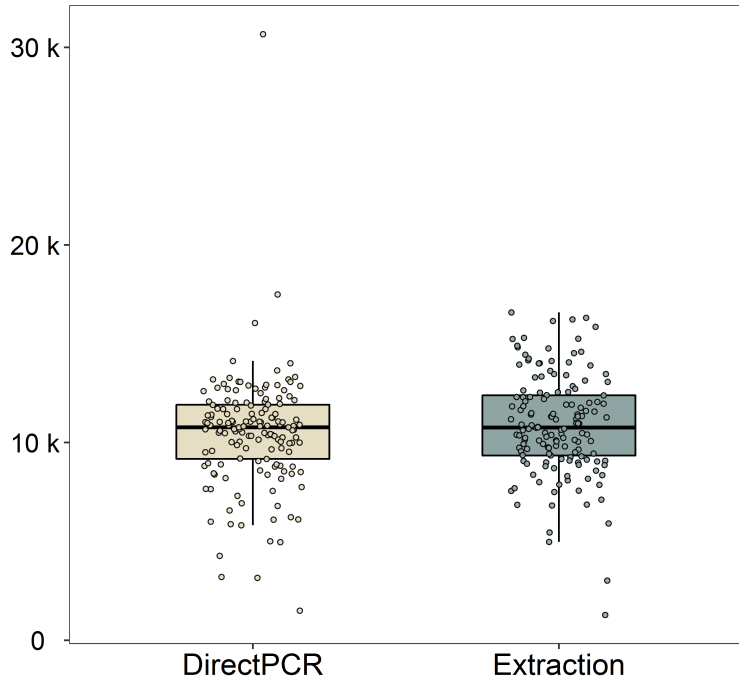

Supplement: FIG S2 [file sys006172153sf4.pdf]

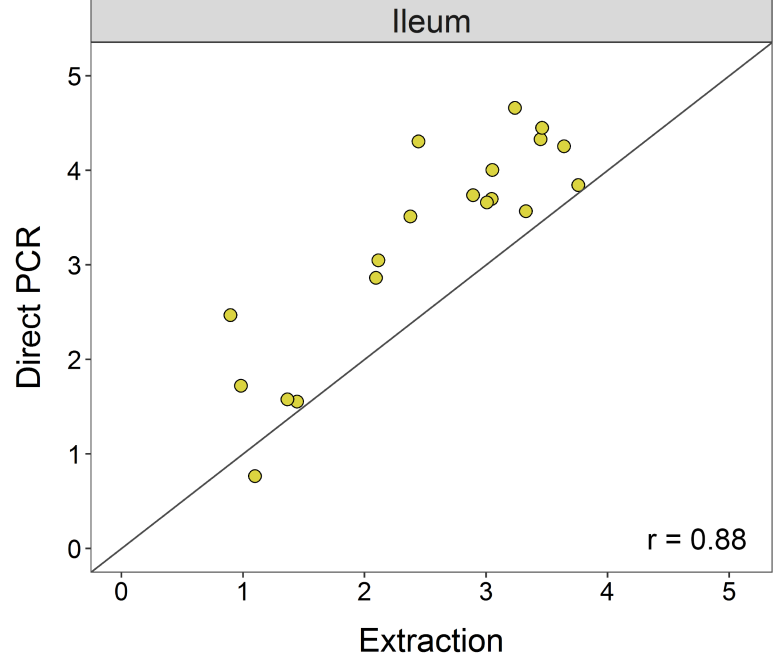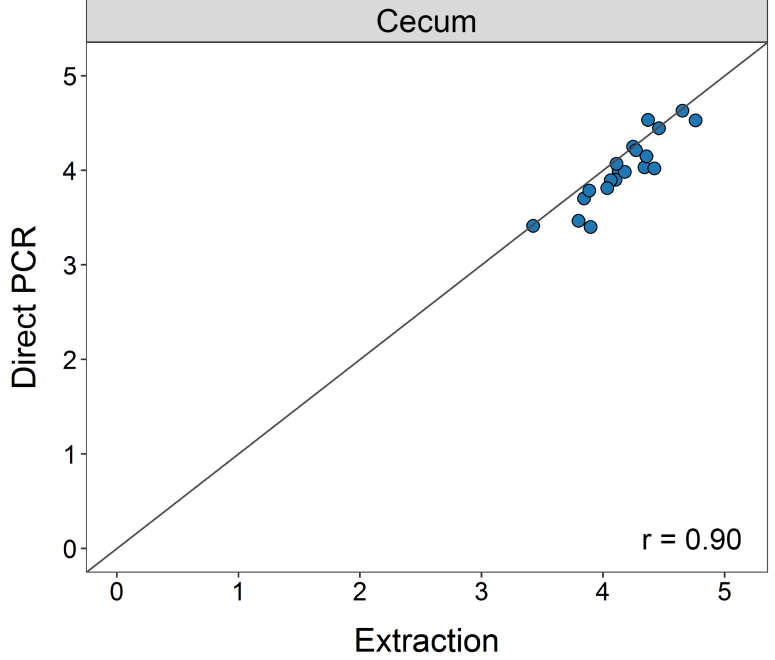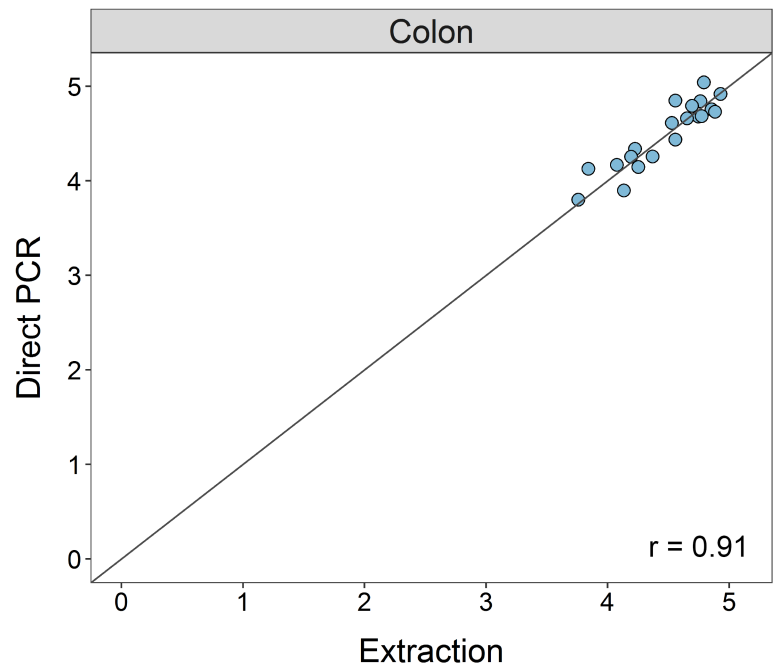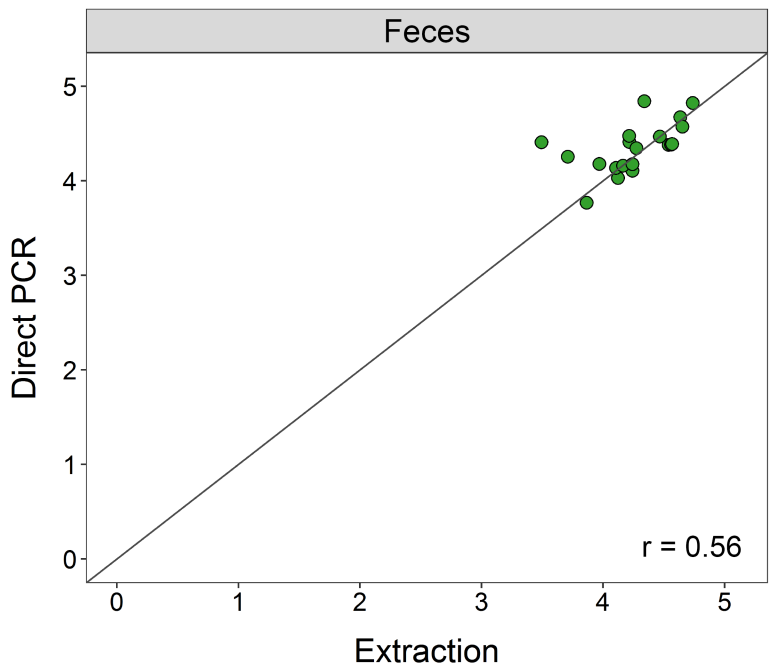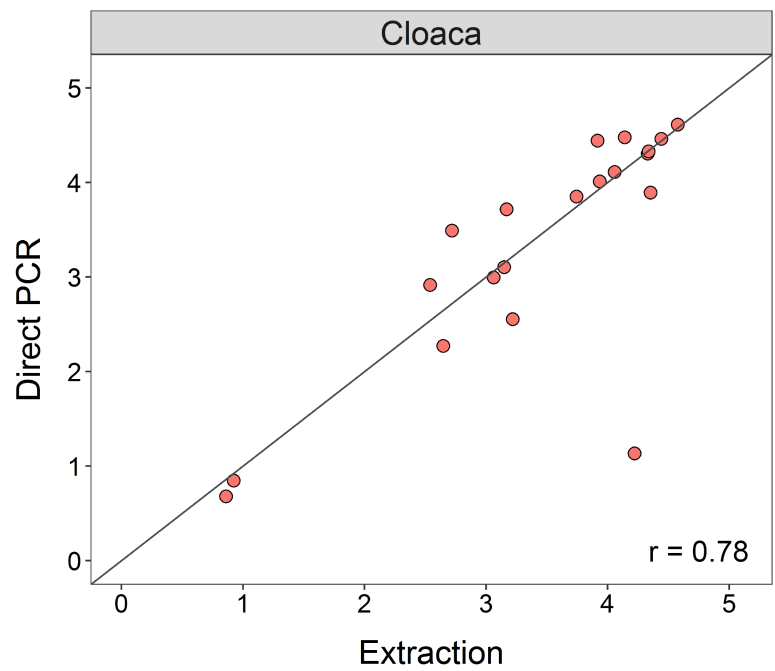

Supplement: FIG S3 [file sys006172153sf5.pdf]

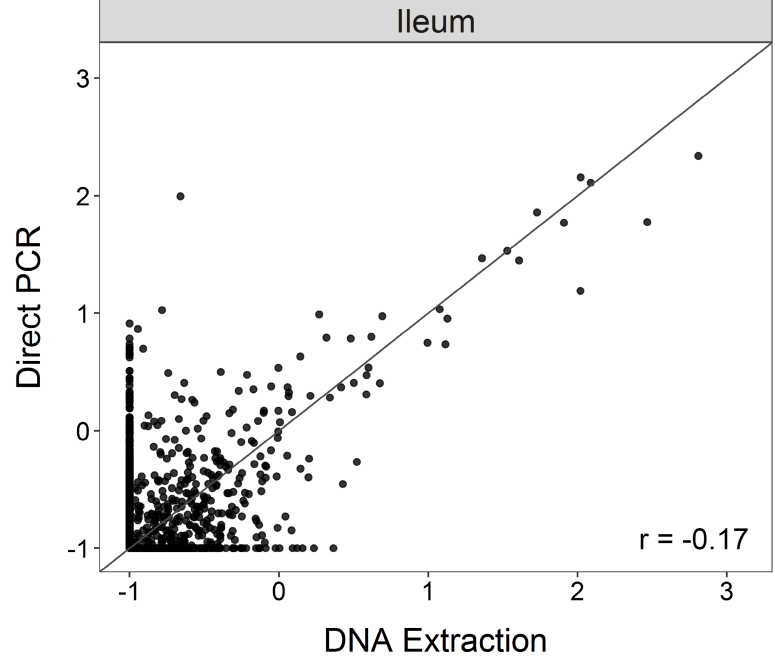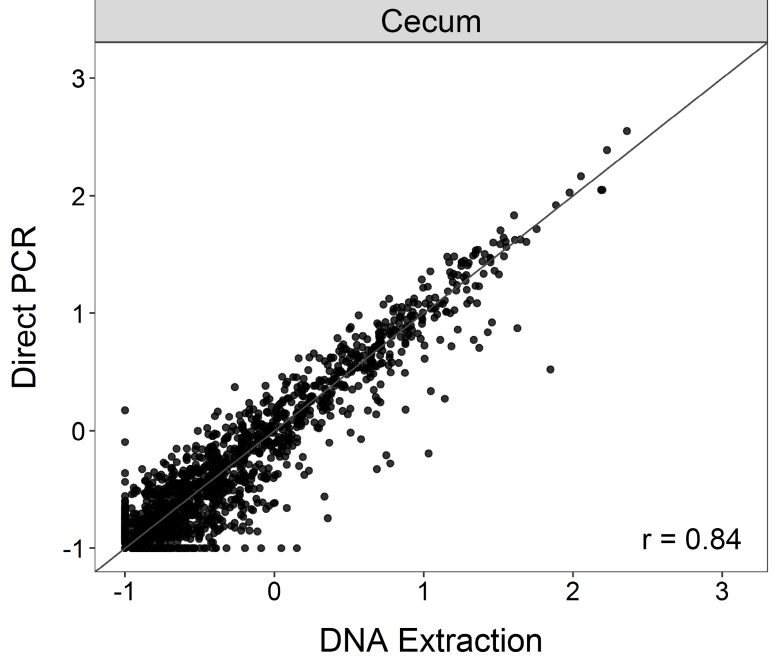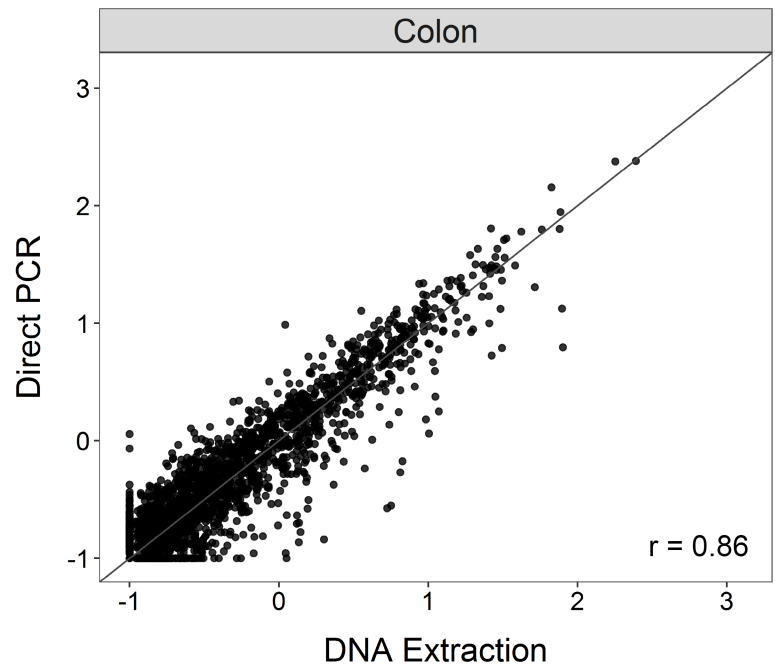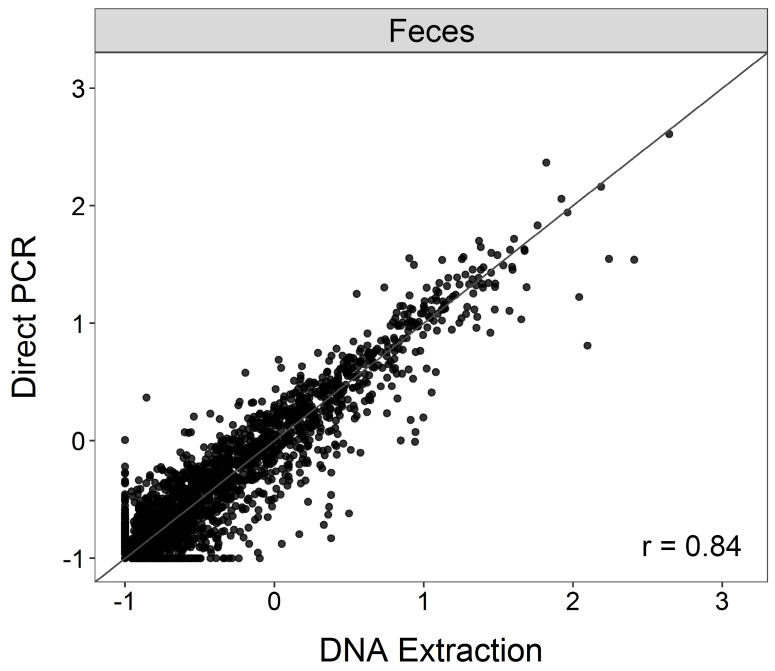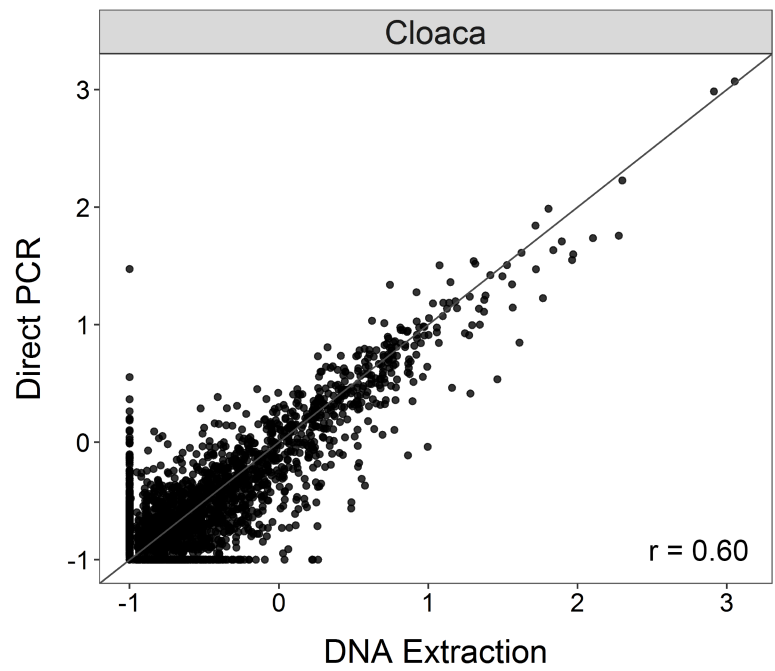

Supplement: FIG S4 [file sys006172153sf6.pdf]

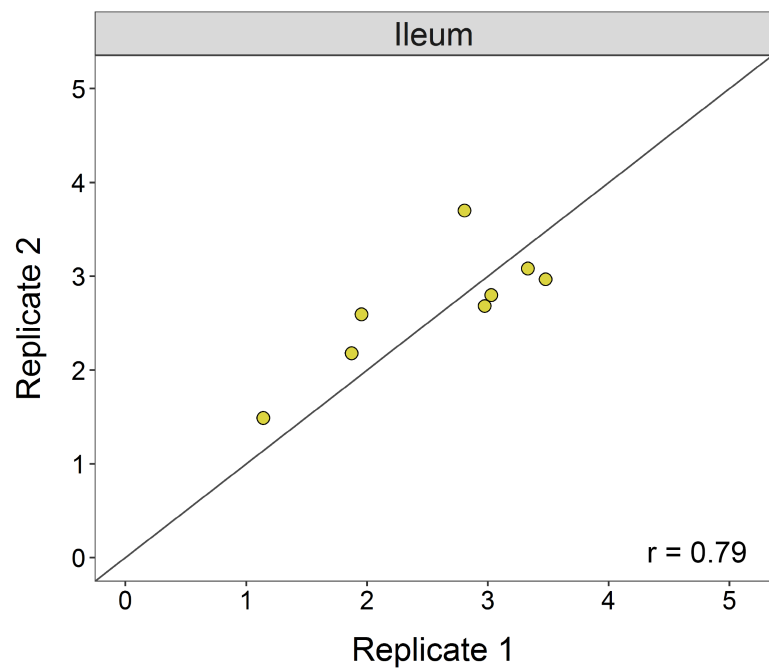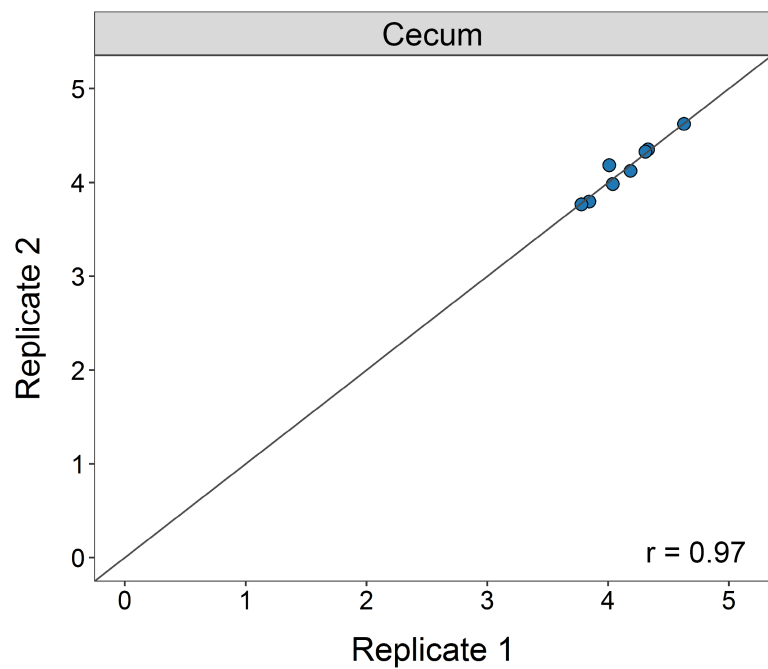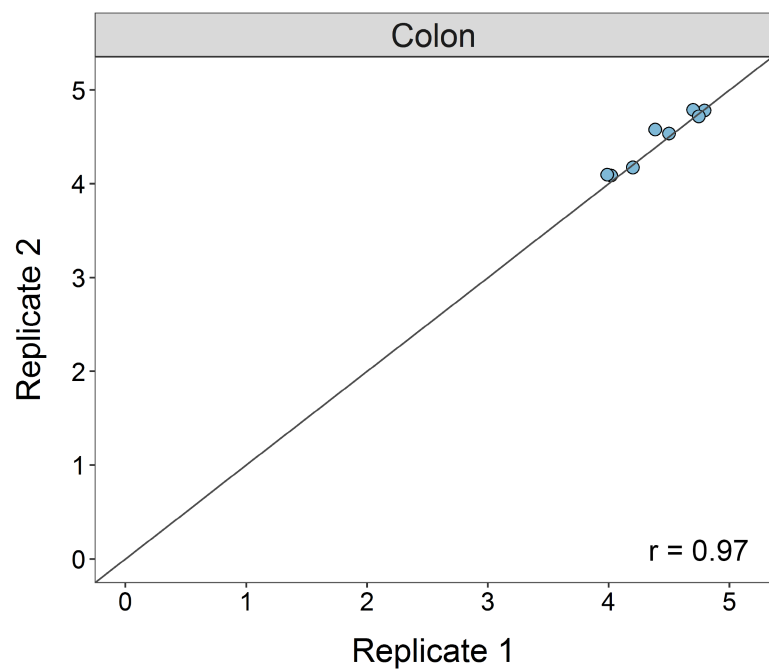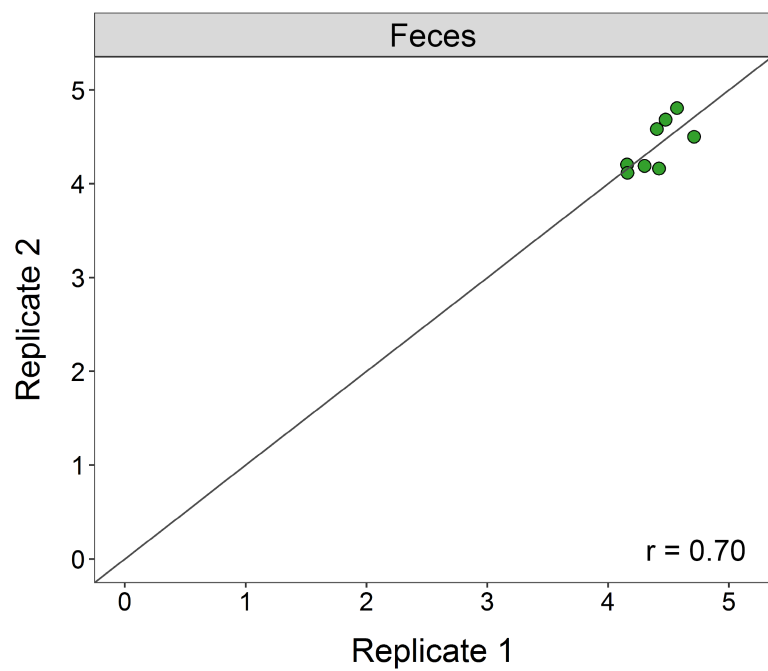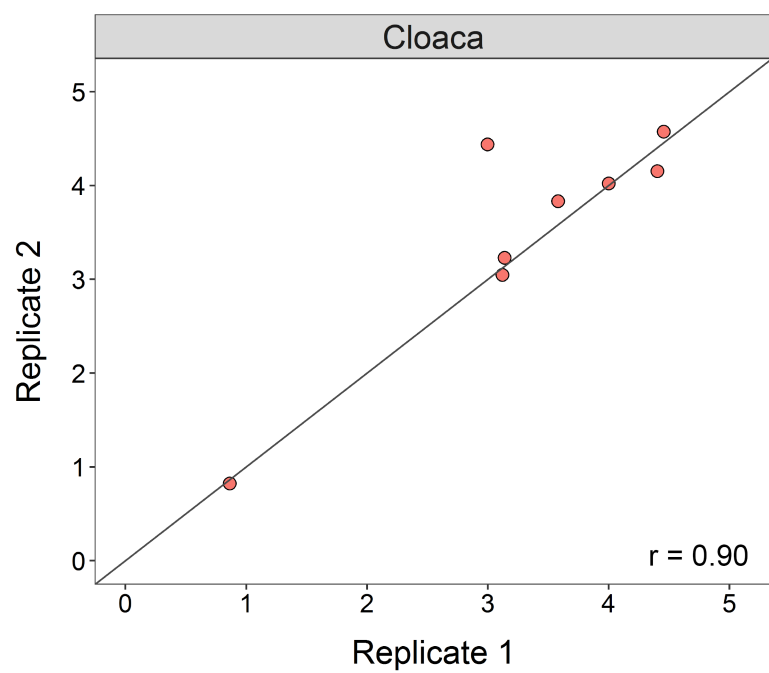

Supplement: FIG S5 [file sys006172153sf7.pdf]

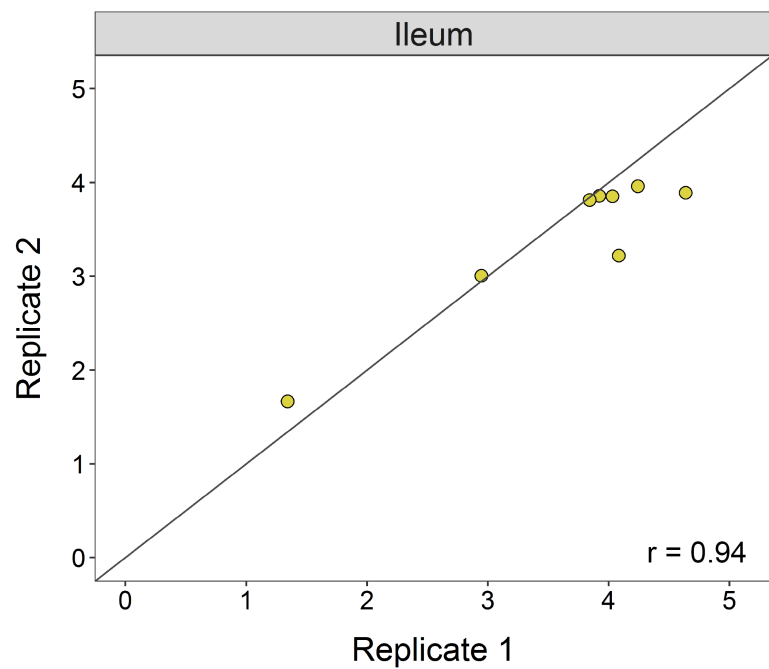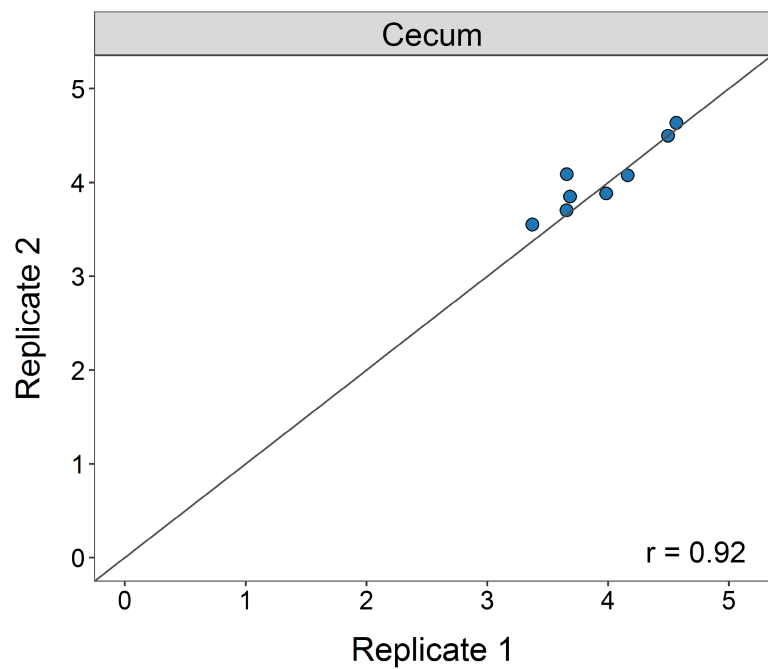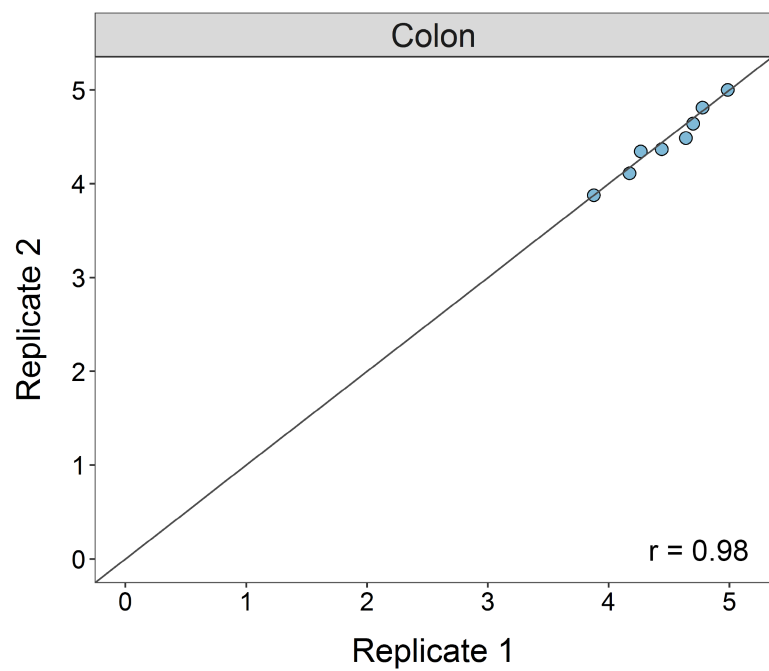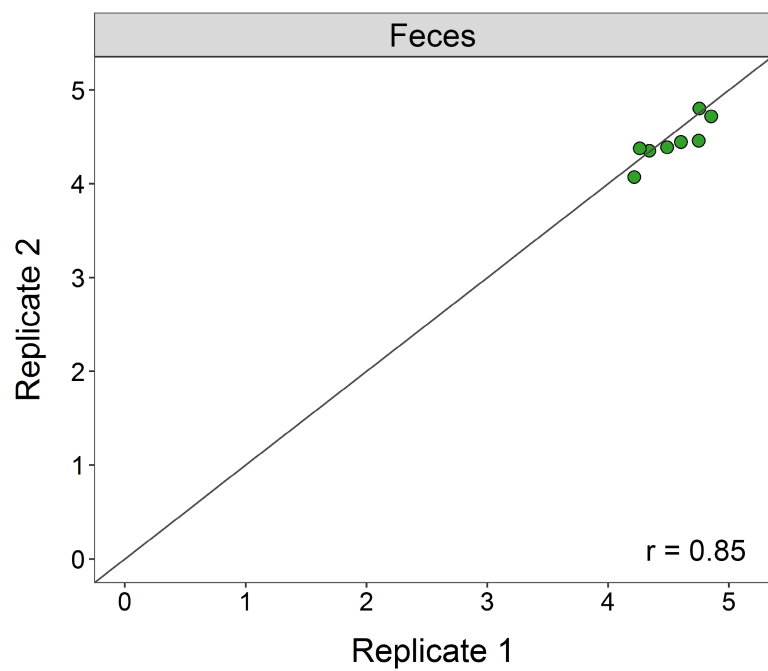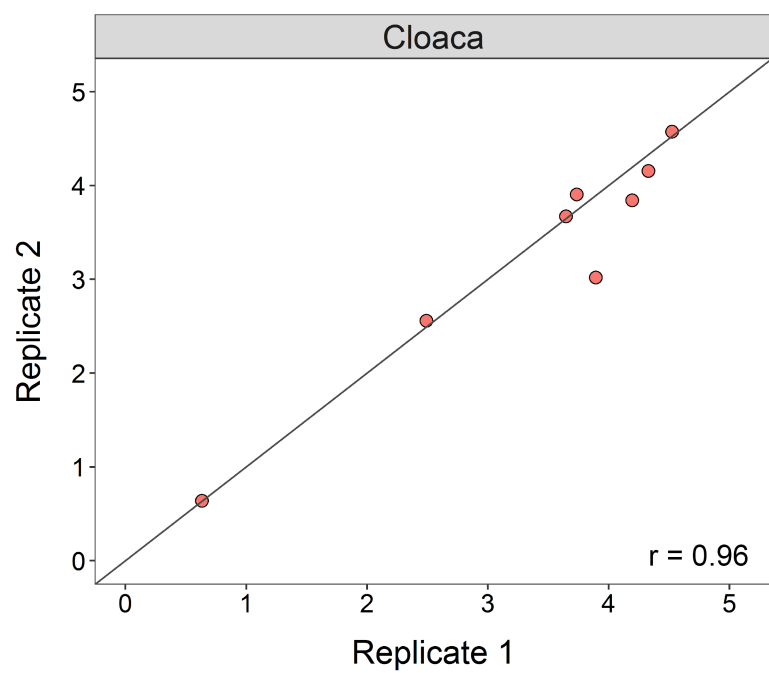

Supplement: FIG S6 [file sys006172153sf8.pdf]
